# Supplementary material for: Association of Coffee and Tea Intake with Bone Mineral Density and Hip Fracture: A Meta-Analysis
Source: Medicina (Kaunas). 2023 Jun 20;59(6):1177. doi: 10.3390/medicina59061177 (PMC10301353; doi:10.3390/medicina59061177)
Supplement: Supplementary file 1 [file medicina-59-01177-s001.zip › medicina-2400979-supplementary.pdf]

medicina

Supplementary Material

Association of Coffee and Tea Intake with Bone Mineral Density and Hip Fracture: A Meta-analysis

Chun-Ching Chen[1, †], Yu-Ming Shen[2, †], Siou-Bi Li[3, †], Shu-Wei Huang[4], Yi-Jie Kuo[5] and Yu-Pin Chen[6,*]

* **Correspondence:** Yu-Pin Chen: 99231@w.tmu.edu.tw

1 Supplementary Figures and Tables

1.1 Supplementary Figures

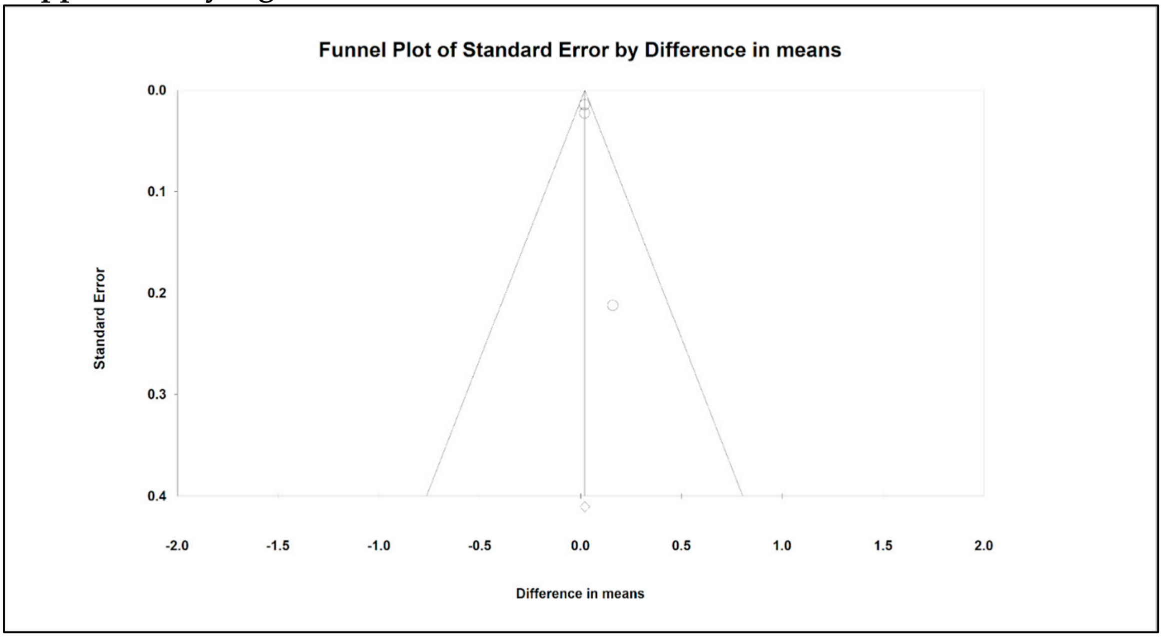

(A)

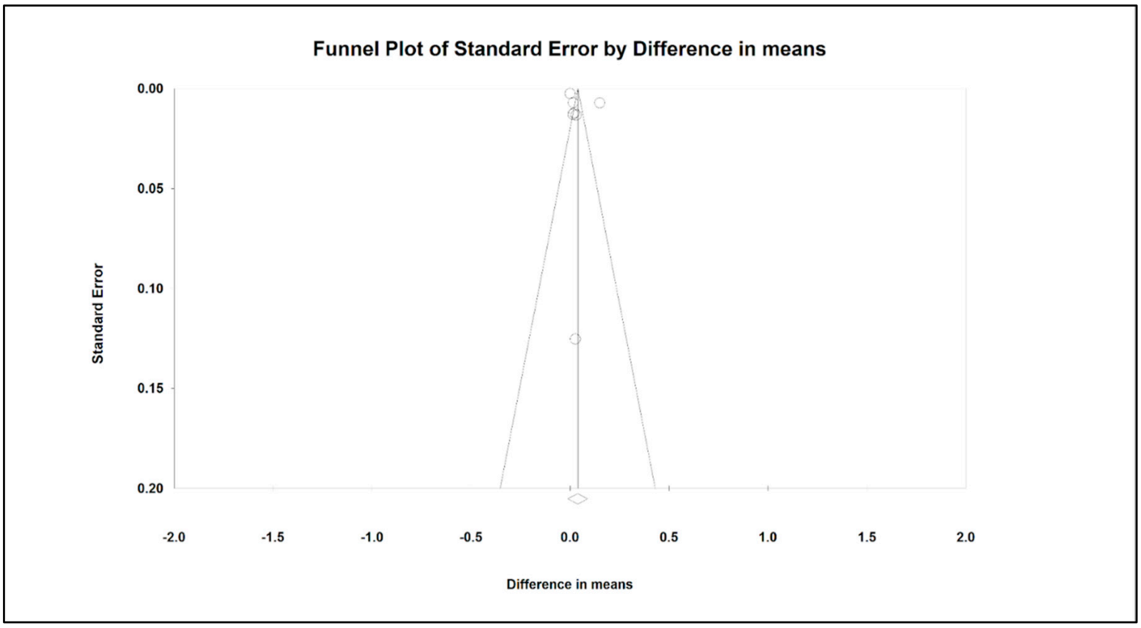

(B)

Figure S1. The funnel plots of the effect of coffee/tea consumption on BMD in the whole body. (A) coffee (B) tea

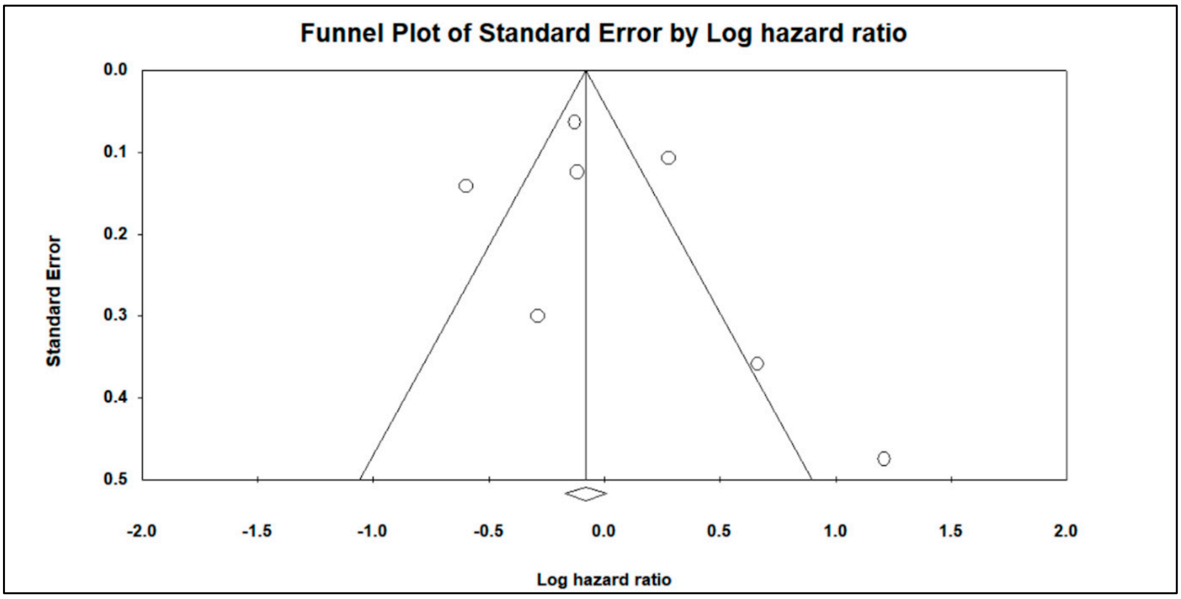

(A)

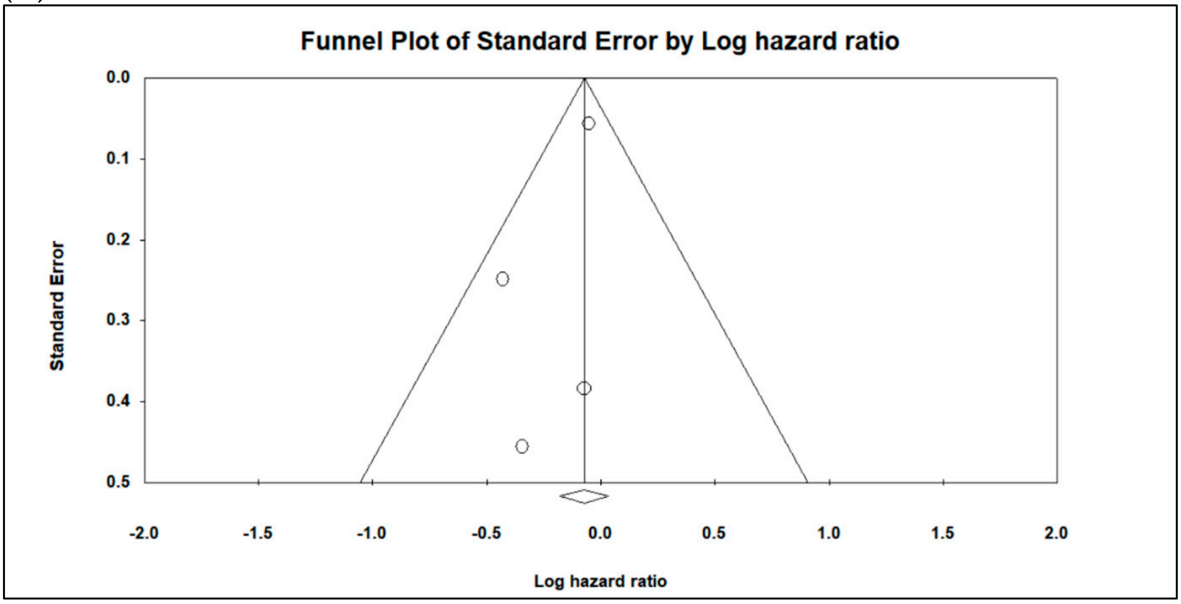

(B)

Figure S2. The funnel plots of the effect of coffee/tea consumption on risk of hip fracture.
(A) coffee (B) tea

1.2 Supplementary tables

1.2.1 Table S1a, S1b. MeSH searching text

coffee

Pubmed
((“Bone Density"[Mesh]) OR "Hip Fractures"[Mesh]) AND "Coffee"[Mesh]
(("Bone Density"[Mesh]) OR "Fractures, Bone"[Mesh]) AND "Coffee"[Mesh]
Embase
('bone density'/exp OR 'bone density' OR 'fracture'/exp OR 'fracture') AND ('coffee'/exp OR 'coffee')
('bone density'/exp OR 'bone density' OR 'hip fracture'/exp OR 'hip fracture') AND ('coffee'/exp OR 'coffee')

Tea

Pubmed
((“Bone Density"[Mesh]) OR "Hip Fractures"[Mesh]) AND "tea"[Mesh]
(("Bone Density"[Mesh]) OR "Fractures, Bone"[Mesh]) AND "tea"[Mesh]
Embase
('bone density'/exp OR 'bone density' OR 'fracture'/exp OR 'fracture') AND ('tea'/exp OR 'tea')
('bone density'/exp OR 'bone density' OR 'hip fracture'/exp OR 'hip fracture') AND ('tea'/exp OR 'tea')

1.2.2 **Table S2.** Standardized data collection form

General Information

Study Characteristics	Review Inclusion Criteria	Yes/ No / Unclear	Location in text (pg & ¶/fig/table)
Type of study	Controlled before-after study		
	Contemporaneous data collection		
	At least 2 intervention and 2 control clusters	...	
	Other design (specify):		
		...	
Participants		...	
Types of intervention		...	
Types of outcome measures		...	
Decision:	...		
Reason for exclusion			
Notes:			

Population and setting

	Description	Location in text<br>(pg & ¶/fig/table)
Population description		
Setting		
Inclusion criteria		
Exclusion criteria		
Method/s of recruitment of participants		
Notes:		

Methods

	Descriptions as stated in report/paper	Location in text<br>(pg & ¶/fig/table)
Aim of study		
Design		
Unit of allocation		
Start date		
End date		

Duration of participation		
Notes:		

Participants

	Description as stated in report/paper	Location in text (pg & ¶/fig/table)
Total no.		
Baseline imbalances		
Withdrawals and exclusions		
Age		
Sex		
Severity of illness		
Co-morbidities		
Other treatment received		
Other relevant sociodemographics		
Subgroups measured		
Subgroups reported		
Notes:		

Intervention groups

Intervention Group 1

	Description as stated in report/paper	Location in text<br><br>(pg & ¶/fig/table)
Group name		
No.		
Description		
Duration of treatment period		
Timing		
Delivery		
Providers		
Co-interventions		
Economic variables		
Resource requirements to replicate intervention		
Notes:		

Outcomes

	Description as stated in report/paper		Location in text<br><br>(pg & ¶/fig/table)
Outcome name			
Time points measured			
Time points reported			
Outcome definition			
Person measuring/ reporting			
Scales: upper and lower limits			
Is outcome/tool validated?	... Yes/No/Unclear		
Imputation of missing data			
Assumed risk estimate			
Notes:			

Results

	Description as stated in report/paper	Location in text<br><br>(pg & ¶/fig/table)
Comparison		

	Description as stated in report/paper				Location in text<br>(pg & ¶/fig/table)
Outcome					
Subgroup					
Timepoint					
Post-intervention or change from baseline?					
Results	Intervention result	SD (or other variance)	Control result	SD (or other variance)	
	Overall results		SE (or other variance)		
No. participants	Intervention		Control		
No. missing participants and reasons					
No. participants moved from other group and reasons					
Any other results reported					

	Description as stated in report/paper	Location in text<br><br>(pg & ¶/fig/table)
Unit of analysis		
Statistical methods used and appropriateness of these methods		

Data organization of BMD:

1.2.3 Table S3. BMD Excluded studies and reasons

**Reasons for exclusion:**	**Amount**	**Reference No.**
Only abstract available or cannot gain the full content of study	5	[1–5]
Insufficient data for analysis	19	[6–24]
BMD measurement not using DXA	10	[25–34]
Grouping mismatching criteria(no exact grouping based on different levels of coffee/tea consumption)	2	[35,36]
Analysis data of dietary consumption only including caffeine (no discrimination between coffee/tea and other caffeinated drinks or other food)	8	[37–44]
Outcome mismatching criteria(no exact BMD data for different consumption groups)	19	[45–63]
Only including specific population	1	[64]

Cutoff point being too inconsistent to be organized within selected studies	3	[65–67]

Data organization of fracture:

1.2.4 Table S4. Fracture excluded studies and reasons

**Reasons for exclusion:**	**Amount**	**Reference No.**
Insufficient data for analysis	1	[68]
Grouping mismatching criteria(no exact grouping based on different levels of coffee/tea consumption)	4	[69–72]
Analysis data of dietary consumption only including caffeine (no discrimination between coffee/tea and other caffeinated drinks or other food)	2	[73,74]
Outcome mismatching criteria(results of study without hazard ratio, cannot converted to hazard ratio, or no recording fractures of hip)	16	[21,75–89]
Cutoff point being too inconsistent to be organized within selected studies	2	[90,91]

1.2.5 Table S5. Quality assessment of cohort studies using NOS

Article	Representativeness of the exposed cohort	Selection of the non exposed cohort	Ascertainment of exposure	Demonstration that outcome of interest was not present at start of study	Comparability of cohorts on the basis of the design or analysis	Assessment of outcome	Was follow-up long enough for outcomes to occur	Adequacy of follow up of cohorts	Total score
Hallström, H. 2010	*	*	NO	NO	**	*	*	NO	6
Huang, H.2018	*	*	*	*	**	*	*	NO	8
Chen, Z. 2003	*	*	NO	*	**	*	*	*	8
Dai, Z.2018	*	*	NO	*	**	*	*	*	8
Hallström, H.2014	*	*	*	*	**	*	*	*	9
Hallström, H.2013	*	*	NO	NO	**	*	*	*	7

van - Lenthe , F. J. 2011	*	*	NO	*	*	*	*	NO	6
Trimpou, P. 2010	*	*	NO	*	**	*	*	NO	7
Meyer, H. E. 1997	*	*	NO	*	*	*	*	*	7
Hernandez-Avila, M. 1991	NO	*	NO	*	**	*	*	*	7
Myers, G. 2015	*	*	NO	*	**	*	*	*	8

1.2.6 Table S6. Quality assessment of cross-sectional studies using JBI

Article	1	2	3	4	5	6	7	8	Total score
Ye, Y. 2021	YES	YES	NO	YES	NO	NO	YES	YES	5
Lloyd, T. 1997	YES	YES	NO	YES	YES	YES	YES	YES	7
Ni, S. 2021	YES	YES	YES	YES	YES	YES	YES	YES	8
Li, J.-Y.2021	YES	YES	YES	YES	YES	YES	YES	YES	8
Wang, G. 2014	YES	YES	NO	YES	YES	YES	YES	YES	7
Devine, A. 2007	YES	YES	NO	YES	YES	YES	YES	YES	7
Hegarty, V. M. 2000	YES	YES	NO	YES	YES	YES	YES	YES	7

Reference

1. *The effect of smoking tobacco and drinking of alcohol and coffee on bone mineral density of healthy men 40 years of age | Request PDF.* DOI: www.researchgate.net/publication/12096146_The_effect_of_smoking_tobacco_and_drinking_of_alcohol_and_coffee_on_bone_mineral_density_of_healthy_men_40_years_of_age (accessed 2021-12-28).

2. Bauer, D. C.; Browner, W. S.; Cauley, J. A.; Orwoll, E. S.; Scott, J. C.; Black, D. M.; Tao, J. L.; Cummings, S. R.; Nevitt, M. C.; Genant, H. K.; Arnaud, C.; Faulkner, K.; Fox, C.; Gluer, C.; Harvey, S.; Hulley, S. B.; Palermo, L.; Seeley, D.; Steiger, P. Factors Associated with Appendicular Bone Mass in Older Women. *Ann Intern Med* **1993**, *118* (9), 657–665. DOI: doi.org/10.7326/0003-4819-118-9-199305010-00001.

3. Trivitayaratana, W.; Bunyaratavej, N.; Trivitayaratana, P.; Kotivongsa, K.; SuphayaAchin, K.; Chongcharoenkamol, T. The Use of Historical and Anthropometric Data as Risk Factors for Screening of Low MBMD & MCI. *JOURNAL OF THE MEDICAL ASSOCIATION OF THAILAND* **2000**, *83* (2), 129.

4. Ramel, A. Coffee Consumption, Bone Mineral Density and Fracture Risk in Icelandic Old Adults. *Osteoporosis International* **2020**, *31* (SUPPL 1), S620. DOI: doi.org/10.1007/s00198-020-05696-3.

5. Xu, L.; Han, Y.; Zhang, Y. H.; Zhang, X. Y.; Fan, C.; Huang, H. T.; Li, P.; Geng, Z. Correlation Analysis of Bone Mineral Density and Static Balance Ability in Postmenopausal Women. *Journal of Investigative Medicine* **2019**, *67*, A1. DOI: doi.org/10.1136/jim-2019-000994.2.

6. Holvik, K.; Meyer, H. E. The Association between Caffeine Intake and Forearm Bone Mineral Density in Postmenopausal Women: The Oslo Health Study. *Norsk Epidemiologi* **2011**, *13* (1). DOI: doi.org/10.5324/NJE.V13I1.326.

7. Demirbag, D.; Ozdemir, F.; Ture, M. Effects of Coffee Consumption and Smoking Habit on Bone Mineral Density. *Rheumatol Int* **2006**, *26* (6), 530–535. DOI: doi.org/10.1007/S00296-005-0020-4/FIGURES/4.

8. Kapetanović, A.; Avdic, D. Influence of Coffee Consumption on Bone Mineral Density in Postmenopausal Women with Estrogen Deficiency in Menstrual History. *Journal of Health Sciences* **2014**, *4* (2), 105–109. DOI: doi.org/10.17532/JHSCI.2014.194.

9. Hannan, M. T.; Felson, D. T.; Dawson-Hughes, B.; Tucker, K. L.; Cupples, L. A.; Wilson, P. W. F.; Kiel, D. P. Risk Factors for Longitudinal Bone Loss in Elderly Men and Women: The Framingham Osteoporosis Study. *J Bone Miner Res* **2000**, *15* (4), 710–720. DOI: doi.org/10.1359/JBMR.2000.15.4.710.

10. Atalar, E.; Aydin, G.; Keles, I.; Inal, E.; Zog, G.; Arslan, A.; Orkun, S. Factors Affecting Bone Mineral Density in Men. *Rheumatol Int* **2009**, *29* (9), 1025–1030. DOI: doi.org/10.1007/S00296-008-0768-4.

11. Je, Y.; Jeong, S.; Park, T. Coffee Consumption Patterns in Korean Adults: The Korean National Health and Nutrition Examination Survey (2001-2011). *Asia Pac J Clin Nutr* **2014**, *23* (4), 691–702. DOI: doi.org/10.6133/APJCN.2014.23.4.11.

12. Whittle, C. R.; Woodside, J. V.; Cardwell, C. R.; McCourt, H. J.; Young, I. S.; Murray, L. J.; Boreham, C. A.; Gallagher, A. M.; Neville, C. E.; McKinley, M. C. Dietary Patterns and Bone Mineral Status in Young Adults: The Northern Ireland Young Hearts Project. *Br J Nutr* **2012**, *108* (8), 1494–1504. DOI: doi.org/10.1017/S0007114511006787.

13. Hardcastle, A. C.; Aucott, L.; Fraser, W. D.; Reid, D. M.; MacDonald, H. M. Dietary Patterns, Bone Resorption and Bone Mineral Density in Early Post-Menopausal Scottish Women. *European Journal of Clinical Nutrition 2011 65:3* **2010**, *65* (3), 378–385. DOI: doi.org/10.1038/ejcn.2010.264.

14. Tucker, K. L.; Chen, H.; Hannan, M. T.; Adrienne Cupples, L.; Wilson, P. W. F.; Felson, D.; Kiel, D. P. Bone Mineral Density and Dietary Patterns in Older Adults: The Framingham Osteoporosis Study. *Am J Clin Nutr* **2002**, *76* (1), 245–252. DOI: doi.org/10.1093/AJCN/76.1.245.

15. Park, S. J.; Joo, S. E.; Min, H.; Park, J. K.; Kim, Y.; Kim, S. S.; Ahn, Y. Dietary Patterns and Osteoporosis Risk in Postmenopausal Korean Women. *Osong Public Health Res Perspect* **2012**, *3* (4), 199–205. DOI: doi.org/10.1016/J.PHRP.2012.10.005.

16. Hansen, M. A.; Overgaard, K.; Riis, B. J.; Christiansen, C. Potential Risk Factors for Development of Postmenopausal Osteoporosis--Examined over a 12-Year Period. *Osteoporos Int* **1991**, *1* (2), 95–102. DOI: doi.org/10.1007/BF01880450.

17. Reid, I. R.; Ames, R. W.; Evans, M. C.; Sharpe, S. J.; Gamble, G. D. Determinants of the Rate of Bone Loss in Normal Postmenopausal Women. *J Clin Endocrinol Metab* **1994**, *79* (4), 950–954. DOI: doi.org/10.1210/JCEM.79.4.7962303.

18. Vestergaard, P.; Hermann, A. P.; Gram, J.; Jensen, L. B.; Eiken, P.; Abrahamsen, B.; Brot, C.; Kolthoff, N.; Sørensen, O. H.; Beck Nielsen, H.; Pors Nielsen, S.; Charles, P.; Mosekilde, L. Evaluation of Methods for Prediction of Bone Mineral Density by Clinical and Biochemical Variables in Perimenopausal Women. *Maturitas* **2001**, *40* (3), 211–220. DOI: doi.org/10.1016/S0378-5122(01)00240-7.

19. Ilich, J. Z.; Cvijetic, S. C.; Baric, I. C.; Cecic, I.; Saric, M.; Crncevic-Orlic, Z.; Blanusa, M.; Korsic, M. Nutrition and Lifestyle in Relation to Bone Health and Body Weight in Croatian Postmenopausal Women. *Int J Food Sci Nutr* **2009**, *60* (4), 319–332. DOI: doi.org/10.1080/09637480701780724.

20. Kalem, M. N.; Kalem, Z.; Akgun, N.; Bakırarar, B. The Relationship between Postmenopausal Women's Sclerostin Levels and Their Bone Density, Age, Body Mass Index, Hormonal Status, and Smoking and Consumption of Coffee and Dairy Products. *Arch Gynecol Obstet* **2017**, *295* (3), 785–793. DOI: doi.org/10.1007/S00404-017-4288-X/TABLES/1.

21. Yuan, S.; Michaëlsson, K.; Wan, Z.; Larsson, S. C. Associations of Smoking and Alcohol and Coffee Intake with Fracture and Bone Mineral Density: A Mendelian Randomization Study. *Calcif Tissue Int* **2019**, *105* (6), 582–588. DOI: doi.org/10.1007/S00223-019-00606-0.

22. Chan, C. Y.; Subramaniam, S.; Chin, K. Y.; Ima-Nirwana, S.; Muhammad, N.; Fairus, A.; Rizal, A. M. M.; Ng, P. Y.; Aini, J. N.; Aziz, N. A.; Mohamed, N. Knowledge, Beliefs, Dietary, and Lifestyle Practices Related to Bone Health among Middle-Aged and Elderly Chinese in Klang Valley, Malaysia. *Int J Environ Res Public Health* **2019**, *16* (10). DOI: doi.org/10.3390/IJERPH16101787.

23. Dietary Pattern-Based Differences in the Associations Between Weight Control, Bone Mineral Density, and the Risk of Fracture Among Iranian Postmenopausal Women With Osteoporosis: An Application of Theory of Planned Behavior.

24. Lee, D. B.; Song, H. J.; Paek, Y. J.; Park, K. H.; Seo, Y. G.; Noh, H. M. Relationship between Regular Green Tea Intake and Osteoporosis in Korean Postmenopausal Women: A Nationwide Study. *Nutrients* **2022**, *14* (1). DOI: doi.org/10.3390/NU14010087.

25. Johansson, C.; Mellstrom, D. Coffee Drinking: A Minor Risk Factor for Bone Loss and Fractures.

26. Cauley, J. A.; Fullman, R. L.; Stone, K. L.; Zmuda, J. M.; Bauer, D. C.; Barrett-Connor, E.; Ensrud, K.; Lau, E. M. C.; Orwoll, E. S. Factors Associated with the Lumbar Spine and Proximal Femur Bone Mineral Density in Older Men. *Osteoporosis International* **2005**, *16* (12), 1525–1537. DOI: doi.org/10.1007/S00198-005-1866-8/TABLES/5.

27. Yang, P.; Zhang, X. Z.; Zhang, K.; Tang, Z. Associations between Frequency of Coffee Consumption and Osteoporosis in Chinese Postmenopausal Women. *Int J Clin Exp Med* **2015**, *8* (9), 15958.

28. Yu, Q.; Liu, Z. H.; Lei, T.; Tang, Z. Subjective Evaluation of the Frequency of Coffee Intake and Relationship to Osteoporosis in Chinese Men. *J Health Popul Nutr* **2016**, *35* (1), 24. DOI: doi.org/10.1186/S41043-016-0060-2/TABLES/5.

29. Al-Ayyadhi, N.; Refaat, L. A. E.; Ibrahim, M. M.; Elgalil, H. M. A. Screening for Bone Mineral Density and Assessment Knowledge Level of Low Peak Bone Risk Factors and Preventive Practices among Kuwaiti Future Mothers. *J Multidiscip Healthc* **2020**, *13*, 1983–1991. DOI: doi.org/10.2147/JMDH.S280261.

30. Chin, K. Y.; Low, N. Y.; Dewiputri, W. I.; Ima-Nirwanaa, S. Factors Associated with Bone Health in Malaysian Middle-Aged and Elderly Women Assessed via Quantitative Ultrasound. *Int J Environ Res Public Health* **2017**, *14* (7). DOI: doi.org/10.3390/IJERPH14070736.

31. Chang, H.-C.; Hsieh, C.-F.; Lin, Y.-C.; Manli Tantoh, D.; Kung, Y.-Y.; Lin, M.-C.; Liaw, Y.-C.; Liaw, Y.-P. Coffee Consumption Might Reduce the Risk of Osteopenia/Osteoporosis in Premenopausal Taiwanese Women. *Journal of Food and Nutrition Research* **2017**, *5* (10), 789–793. DOI: doi.org/10.12691/JFNR-5-10-10.

32. Chang, H. C.; Hsieh, C. F.; Lin, Y. C.; Tantoh, D. M.; Ko, P. C.; Kung, Y. Y.; Wang, M. C.; Hsu, S. Y.; Liaw, Y. C.; Liaw, Y. P. Does Coffee Drinking Have Beneficial Effects on Bone Health of Taiwanese Adults? A Longitudinal Study. *BMC Public Health* **2018**, *18* (1), 1–10. DOI: doi.org/10.1186/S12889-018-6168-0/TABLES/5.

33. Keskin, Y.; Çekin, M. D.; Gündüz, H.; Lüleci, N. E.; Giray, E.; Sur, H.; Akyüz, G. The Prevalence of Osteoporosis in the Thrace Region of Turkey: A Community-Based Study. *Turkiye Fiziksel Tip ve Rehabilitasyon Dergisi* **2014**, *60* (4), 335–340. DOI: doi.org/10.5152/TFTRD.2014.34033.

34. Forsmo, S.; Schei, B.; Langhammer, A.; Forsén, L. How Do Reproductive and Lifestyle Factors Influence Bone Density in Distal and Ultradistal Radius of Early Postmenopausal Women? The Nord-Trøndelag Health Survey, Norway. *Osteoporosis International 2001 12:3* **2001**, *12* (3), 222–229. DOI: doi.org/10.1007/S001980170133.

35. Berriche, O.; Chiraz, A.; Othman, R. Ben; Souheila, H.; Lahmer, I.; Wafa, C.; Sebai, I.; Sfar, H.; Mahjoub, F.; Jamoussi, H.; Journal, A. Nutritional Risk Factors for Postmenopausal Osteoporosis. *Alexandria Journal of Medicine* **2016**. DOI: doi.org/10.1016/j.ajme.2016.03.007.

36. Franceschi, S.; Schinella, D.; Bidoli, E.; Dal Maso, L.; Vecchia, C. La; Parazzini, F.; Zecchin, R. The Influence of Body Size, Smoking, and Diet on Bone Density in Pre-and Postmenopausal. *Source: Epidemiology* **1996**, *7* (4), 411–414.

37. Huitrón-Bravo, G.; Denova-Gutiérrez, E.; Talavera, J. O.; Moran-Villota, C.; Tamayo, J.; Omaña-Covarrubias, A.; Salmerón, J. Levels of Serum Estradiol and Lifestyle Factors Related with Bone Mineral Density in Premenopausal Mexican Women: A Cross-Sectional Analysis. *BMC Musculoskelet Disord* **2016**, *17* (1). DOI: doi.org/10.1186/S12891-016-1273-7.

38. Conlisk, A. J.; Galuska, D. A. Is Caffeine Associated with Bone Mineral Density in Young Adult Women? *Prev Med (Baltim)* **2000**, *31* (5), 562–568. DOI: doi.org/10.1006/PMED.2000.0742.

39. Hernández-Avila, M.; Stampfer, M. J.; Ravnikar, V. A.; Willett, W. C.; Schiff, I.; Francis, M.; Longscope, C.; McKinlay, S. M. Caffeine and Other Predictors of Bone Density among Pre- and Perimenopausal Women. *Epidemiology* **1993**, *4* (2), 128–134. DOI: doi.org/10.1097/00001648-199303000-00008.

40. De França, N. A. G.; Camargo, M. B. R.; Lazaretti-Castro, M.; Peters, B. S. E.; Martini, L. A. Dietary Patterns and Bone Mineral Density in Brazilian Postmenopausal Women with Osteoporosis: A Cross-Sectional Study. *European Journal of Clinical Nutrition 2016 70:1* **2015**, *70* (1), 85–90. DOI: doi.org/10.1038/ejcn.2015.27.

41. Rapuri, P. B.; Gallagher, J. C.; Kinyamu, H. K.; Ryschon, K. L. Caffeine Intake Increases the Rate of Bone Loss in Elderly Women and Interacts with Vitamin D Receptor Genotypes. *Am J Clin Nutr* **2001**, *74* (5), 694–700. DOI: doi.org/10.1093/AJCN/74.5.694.

42. Lloyd, T.; Johnson-Rollings, N.; Mauger, E. A.; Cusatis, D. C.; Eggli, D. F.; Kieselhorst, K. Bone Status among Postmenopausal Women with Different Habitual Caffeine Intakes: A Longitudinal Investigation. *J Am Coll Nutr* **2000**, *19* (2), 256–261. DOI: doi.org/10.1080/07315724.2000.10718924.

43. Harris, S. S.; Dawson-Hughes, B. Caffeine and Bone Loss in Healthy Postmenopausal Women1-4. *Am J Cliii Nuir* **1994**, *60*, 573–581.

44. Hirata, H.; Kitamura, K.; Saito, T.; Kobayashi, R.; Iwasaki, M.; Yoshihara, A.; Watanabe, Y.; Oshiki, R.; Nishiwaki, T.; Nakamura, K. Association between Dietary Intake and Bone Mineral Density in Japanese Postmenopausal Women: The Yokogoshi Cohort Study. *Tohoku J Exp Med* **2016**, *239* (2), 95–101. DOI: doi.org/10.1620/TJEM.239.95.

45. Hsiao, M. C.; Liu, C. Y.; Wang, C. J. Factors Associated with Low Bone Density among Women with Major Depressive Disorder. *Int J Psychiatry Med* **2012**, *44* (1), 77–90. DOI: doi.org/10.2190/PM.44.1.f.

46. Saitoglu, M.; Ardicoglu, O.; Ozgocmen, S.; Kamanli, A.; Kaya, A. Osteoporosis Risk Factors and Association with Somatotypes in Males. *Arch Med Res* **2007**, *38* (7), 746–751. DOI: doi.org/10.1016/J.ARCMED.2007.03.009.

47. Choi, E. J.; Kim, K. H.; Koh, Y. J.; Lee, J. S.; Lee, D. R.; Park, S. M. Coffee Consumption and Bone Mineral Density in Korean Premenopausal Women. *Korean J Fam Med* **2014**, *35* (1), 11–18. DOI: doi.org/10.4082/KJFM.2014.35.1.11.

48. Barger-Lux, M. J.; Heaney, R. P.; Stegman, M. R. Effects of Moderate Caffeine Intake on the Calcium Economy of Premenopausal Women. *Am J Clin Nutr* **1990**, *52* (4), 722–725. DOI: doi.org/10.1093/AJCN/52.4.722.

49. Zagórski, J. *OSTEOPOROSIS RISK FACTORS IN RURAL AND URBAN WOMEN FROM THE LUBLIN REGION OF POLAND 5DIDá6)LOLS*; 2005; Vol. 12.

50. Korpelainen, R.; Korpelainen, J.; Heikkinen, J.; Väänänen, K.; Keinänen-Kiukaanniemi, S. Lifestyle Factors Are Associated with Osteoporosis in Lean Women but Not in Normal and Overweight Women: A Population-Based Cohort Study of 1222 Women. *Osteoporosis International 2003 14:1* **2003**, *14* (1), 34–43. DOI: doi.org/10.1007/S00198-002-1319-6.

51. Bijelic, R.; Milicevic, S.; Balaban, J. Risk Factors for Osteoporosis in Postmenopausal Women. *Medical Archives* **2017**, *71* (1), 25. DOI: doi.org/10.5455/MEDARH.2017.71.25-28.

52. Cooper, C.; Atkinson, E. J.; Wahner, H. W.; O'Fallon, W. M.; Riggs, B. L.; Judd, H. L.; Melton, L. J. Is Caffeine Consumption a Risk Factor for Osteoporosis? *Journal of Bone and Mineral Research* **1992**, *7* (4), 465–471. DOI: doi.org/10.1002/JBMR.5650070415.

53. Marci, C. D.; Viechnicki, M. B.; Greenspan, S. L. Bone Mineral Densitometry Substantially Influences Health-Related Behaviors of Postmenopausal Women. *Calcified Tissue International 2000 66:2* **2000**, *66* (2), 113–118. DOI: doi.org/10.1007/S002230010024.

54. Kara, I. H.; Aydin, S.; Gemalmaz, A.; Aktürk, Z.; Yaman, H.; Bozdemir, N.; Kurdak, H.; Sitmapinar, K.; Sencar, I. D.; Başak, O.; Akdeniz, M.; Işildar, H.; Burgut, E.; Özcan, S.; Akça, Ü.; Dağdeviren, N.; Ungan, M. Habitual Tea Drinking and Bone Mineral Density in Postmenopausal Turkish Women: Investigation of Prevalence of Postmenopausal Osteoporosis in Turkey (IPPOT Study). *http://dx.doi.org/10.1024/0300-9831.77.6.389* **2013**, *77* (6), 389–397. DOI: doi.org/10.1024/0300-9831.77.6.389.

55. Hallström, H.; Byberg, L.; Glynn, A.; Lemming, E. W.; Wolk, A.; Michaëlsson, K. Long-Term Coffee Consumption in Relation to Fracture Risk and Bone Mineral Density in Women. *Am J Epidemiol* **2013**, *178* (6), 898–909. DOI: doi.org/10.1093/AJE/KWT062.

56. Choi, E.; Choi, K. H.; Park, S. M.; Shin, D.; Joh, H. K.; Cho, E. The Benefit of Bone Health by Drinking Coffee among Korean Postmenopausal Women: A Cross-Sectional Analysis of the Fourth & Fifth Korea National Health and Nutrition Examination Surveys. *PLoS One* **2016**, *11* (1). DOI: doi.org/10.1371/JOURNAL.PONE.0147762.

57. Alshanbari, M. F.; Alsofyani, E. M.; Almalki, D. M.; Alswat, K. A. Caffeine Effect on Bone Mineral Density: A Cross-Sectional Study. *Journal of Clinical and Diagnostic Research* **2018**, *12* (2), OC14–OC17. DOI: doi.org/10.7860/JCDR/2018/31216.11174.

58. Chan, P. J.; Nurul, Z. Z.; Chuah, J. S.; Nabil, M. M. A.; Isa, N. M.; Sabarul, A. M.; Nazrun, A. S. Association between Risk Factors of Osteoporosis and Bone Mineral Density in Women of Different Ethnic Groups in a Malaysian Hospital. *International Journal of Osteoporosis and Metabolic Disorders* **2014**, *7* (1), 1–11. DOI: doi.org/10.3923/IJOM.2014.1.11.

59. Wang, S.; Lin, S.; Zhou, Y.; Wang, Z. Social and Behavior Factors Related to Aged Chinese Women with Osteoporosis. *http://dx.doi.org/10.1080/09513590802309196* **2009**, *24* (10), 538–545. DOI: doi.org/10.1080/09513590802309196.

60. Wang, Y.; Ding, H.; Wang, X.; Wei, Z.; Feng, S. Associated Factors for Osteoporosis and Fracture in Chinese Elderly. *Med Sci Monit* **2019**, *25*, 5580–5588. DOI: doi.org/10.12659/MSM.914182.

61. Palomeras-Vilches, A.; Viñals-Mayolas, E.; Bou-Mias, C.; Jordà-Castro, M.; Agüero-Martínez, M.; Busquets-Barceló, M.; Pujol-Busquets, G.; Carrion, C.; Bosque-Prous, M.; Serra-Majem, L.; Bach-Faig, A. Adherence to the Mediterranean Diet and Bone Fracture Risk in Middle-Aged Women: A Case Control Study. *Nutrients* **2019**, *11* (10). DOI: doi.org/10.3390/NU11102508.

62. Alquaiz, A. M.; Kazi, A.; Tayel, S.; Shaikh, S. A.; Al-Sharif, A.; Othman, S.; Habib, F.; Fouda, M.; Sulaimani, R. Prevalence and Factors Associated with Low Bone Mineral Density in Saudi Women: A Community Based Survey. *BMC Musculoskelet Disord* **2014**, *15* (1). DOI: doi.org/10.1186/1471-2474-15-5.

63. Romero, A. A.; Duarte-Gardea, M.; Ortiz, M.; Labrado, C.; Noe, M. Bone Mineral Density and Body Mass Index of Mexican American Women. *Hispanic Healthcare International* **2005**, *3* (1), 9–14.

64. Grzegorzewska, A. E.; Mlot-Michalska, M. Coffee Consumption and Bone Mineral Density in Dialysis Patients. *Advances in Peritoneal Dialysis* **2008**, *24*.

65. Muraki, S.; Yamamoto, S.; Ishibashi, H.; Oka, H.; Yoshimura, N.; Kawaguchi, H.; Nakamura, K. Diet and Lifestyle Associated with Increased Bone Mineral Density: Cross-Sectional Study of Japanese Elderly Women at an Osteoporosis Outpatient Clinic. *Journal of Orthopaedic Science 2007 12:4* **2007**, *12* (4), 317–320. DOI: doi.org/10.1007/S00776-007-1143-0.

66. Hossein-Nezhad, A.; Maghbooli, Z.; Shafaei, A. R.; Javadi, E.; Larijani, B. Relationship between Tea Drinking and Bone Mineral Density in Iranian Population. *Iran J Public Health* **2007**, *36* (Supple 1), 57–62.

67. Barrett Connor, E.; Chang, J. C.; Edelstein, S. L. Coffee-Associated Osteoporosis Offset by Daily Milk Consumption: The Rancho Bernardo Study. *JAMA* **1994**, *271* (4), 280–283. DOI: doi.org/10.1001/JAMA.1994.03510280042030.

68. Fujiwara, S.; Kasagi, F.; Yamada, M.; Kodama, K. Risk Factors for Hip Fracture in a Japanese Cohort. *undefined* **1997**, *12* (7), 998–1004. DOI: doi.org/10.1359/JBMR.1997.12.7.998.

69. Cummings, S. R.; Nevitt, M. C.; Browner, W. S.; Stone, K.; Fox, K. M.; Ensrud, K. E.; Cauley, J.; Black, D.; Vogt, T. M. Risk Factors for Hip Fracture in White Women. Study of Osteoporotic Fractures Research Group. *N Engl J Med* **1995**, *332* (12), 767–773. DOI: doi.org/10.1056/NEJM199503233321202.

70. Albrand, G.; Munoz, F.; Sornay-Rendu, E.; DuBoeuf, F.; Delmas, P. D. Independent Predictors of All Osteoporosis-Related Fractures in Healthy Postmenopausal Women: The OFELY Study. *Bone* **2003**, *32* (1), 78–85. DOI: doi.org/10.1016/S8756-3282(02)00919-5.

71. Huopio, J.; Kröger, H.; Honkanen, R.; Saarikoski, S.; Alhava, E. Risk Factors for Perimenopausal Fractures: A Prospective Study. *Osteoporosis International 2000 11:3* **2000**, *11* (3), 219–227. DOI: doi.org/10.1007/S001980050284.

72. Ramalho, A. C.; Lazaretti-Castro, M.; Hauache, O.; Vieira, J. G.; Takata, E.; Cafalli, F.; Tavares, F. Osteoporotic Fractures of Proximal Femur: Clinical and Epidemiological Features in a Population of the City of São Paulo. *Sao Paulo Med J* **2001**, *119* (2), 48–53. DOI: doi.org/10.1590/S1516-31802001000200002.

73. Kiel, D. P.; Felson, D. T.; Hannan, M. T.; Anderson, J. J.; F Wilson, P. W.; Felson, T.; Hannan, M. T.; Anderson, J. J.; F Wilson, P. W. CAFFEINE AND THE RISK OF HIP FRACTURE: THE FRAMINGHAM STUDY. *Am J Epidemiol* **1990**, *132* (4), 675–684. DOI: doi.org/10.1093/OXFORDJOURNALS.AJE.A115709.

74. Li, J. Y.; Ge, J. R.; Chen, J.; Ye, Y. J.; Xie, L. H.; Li, L.; Chai, H. Clinical Study on the Influence of Tea Drinking Habits on Bone Mineral Density and Osteoporosis in Postmenopausal Women in Fuzhou City, China. *Progress in Nutrition* **2021**, *23* (1), e2021033–e2021033. DOI: doi.org/10.23751/PN.V23I1.9409.

75. Tavani, A.; Negri, E.; Vecchia, C. La. Coffee Intake and Risk of Hip Fracture in Women in Northern Italy. *Prev Med (Baltim)* **1995**, *24* (4), 396–400. DOI: doi.org/10.1006/pmed.1995.1064.

76. Ma, C. C.; Burchfiel, C. M.; Grove, J.; Fekedulegn, D.; Lu, Y.; Andrew, M. E.; Willcox, B.; Masaki, K. H.; Curb, J. D.; Rodriguez, B. L. Risk Factors for Fractures among Japanese-American Men: The Honolulu Heart Program and Honolulu-Asia Aging Study. *Arch Osteoporos* **2011**, *6* (1–2), 197. DOI: doi.org/10.1007/S11657-011-0068-6.

77. Jha, R. M.; Mithal, A.; Malhotra, N.; Brown, E. M. Pilot Case-Control Investigation of Risk Factors for Hip Fractures in the Urban Indian Population. *BMC Musculoskelet Disord* **2010**, *11* (1), 1–10. DOI: doi.org/10.1186/1471-2474-11-49/TABLES/3.

78. Kreiger, N.; Gross, A.; Hunter, G. Dietary Factors and Fracture in Postmenopausal Women: A Case-Control Study. *Int J Epidemiol* **1992**, *21* (5), 953–958. DOI: doi.org/10.1093/IJE/21.5.953.

79. Suzuki, T.; Yoshida, H.; Hashimoto, T.; Yoshimura, N.; Fujiwara, S.; Fukunaga, M.; Nakamura, T.; Yoh, K.; Inoue, T.; Hosoi, T.; Orimo, H. Case-Control Study of Risk Factors for Hip Fractures in the Japanese Elderly by a Mediterranean Osteoporosis Study (MEDOS) Questionnaire. *Bone* **1997**, *21* (5), 461–467. DOI: doi.org/10.1016/S8756-3282(97)00179-8.

80. Johnell, O.; Gullberg, B.; Kanis, J. A.; Allander, E.; Elffors, L.; Dequeker, J.; Dilsen, G.; Gennari, C.; Vaz, L. A.; Lyritis, G.; Mazzuoli, G.; Miravet, L.; Passeri, M.; Cano, P. R.; Rapado, A.; Ribot, C. Risk Factors for Hip Fracture in European Women: The MEDOS Study. *Journal of Bone and Mineral Research* **1995**, *10* (11), 1802–1815. DOI: doi.org/10.1002/JBMR.5650101125.

81. Kanis, J.; Johnell, O.; Gullberg, B.; Allander, E.; Elffors, L.; Ranstam, J.; Dequeker, J.; Dilsen, G.; Gennari, C.; Lopes Vaz, A.; Lyritis, G.; Mazzuoli, G.; Miravet, L.; Passeri, M.; Perez Cano, R.; Rapado, A.; Ribot, C. Risk Factors for Hip Fracture in Men from Southern Europe: The MEDOS Study. *Osteoporosis International 1999 9:1* **1999**, *9* (1), 45–54. DOI: doi.org/10.1007/S001980050115.

82. Ng, T. P.; Aung, K. C. Y.; Feng, L.; Feng, L.; Nyunt, M. S. Z.; Yap, K. B. Tea Consumption and Physical Function in Older Adults: A Cross-Sectional Study. *The journal of nutrition, health & aging 2014 18:2* **2013**, *18* (2), 161–166. DOI: doi.org/10.1007/S12603-013-0354-7.

83. Cumming, R. G.; Klineberg, R. J. Case-Control Study of Risk Factors for Hip Fractures in the Elderly. *Am J Epidemiol* **1994**, *139* (5), 493–503. DOI: doi.org/10.1093/OXFORDJOURNALS.AJE.A117032.

84. Nieves, J. W.; Grisso, J. A.; Kelsey, J. L. A Case-Control Study of Hip Fracture: Evaluation of Selected Dietary Variables and Teenage Physical Activity. *Osteoporos Int* **1992**, *2* (3), 122–127. DOI: doi.org/10.1007/BF01623818.

85. Jokinen, H.; Pulkkinen, P.; Korpelainen, J.; Heikkinen, J.; Keinänen-Kiukaanniemi, S.; Jämsä, T.; Korpelainen, R. Risk Factors for Cervical and Trochanteric Hip Fractures in Elderly Women: A Population-Based 10-Year Follow-up Study. *Calcif Tissue Int* **2010**, *87* (1), 44–51. DOI: doi.org/10.1007/S00223-010-9382-Z/TABLES/3.

86. van Geel, A. C. M.; Geusens, P. P.; Nagtzaam, I. F.; Schreurs, C. M. J. R.; van der Voort, D. J. M.; Rinkens, P. E. L. M.; Kester, A. D. M.; Dinant, G. J. Timing and Risk Factors for Clinical Fractures among Postmenopausal Women: A 5-Year Prospective Study. *BMC Med* **2006**, *4*, 24. DOI: doi.org/10.1186/1741-7015-4-24.

87. Hallström, H.; Wolk, A.; Glynn, A.; Michaëlsson, K. Coffee, Tea and Caffeine Consumption in Relation to Osteoporotic Fracture Risk in a Cohort of Swedish Women. *Osteoporosis International* **2006**, *17* (7), 1055–1064. DOI: doi.org/10.1007/S00198-006-0109-Y/FIGURES/1.

88. Hansen, S. A.; Folsom, A. R.; Kushi, L. H.; Sellers, T. A. Association of Fractures with Caffeine and Alcohol in Postmenopausal Women: The Iowa Women's Health Study. *Public Health Nutr* **2000**, *3* (3), 253–261. DOI: doi.org/10.1017/S136898000000029X.

89. Trimpou, P.; Odén, A.; Simonsson, T.; Wilhelmsen, L.; Landin-Wilhelmsen, K. High Serum Total Cholesterol Is a Long-Term Cause of Osteoporotic Fracture. *Osteoporos Int* **2011**, *22* (5), 1615–1620. DOI: doi.org/10.1007/S00198-010-1367-2.

90. Chau, Y. P.; Au, P. C. M.; Li, G. H. Y.; Sing, C. W.; Cheng, V. K. F.; Tan, K. C. B.; Kung, A. W. C.; Cheung, C. L. Serum Metabolome of Coffee Consumption and Its Association With Bone Mineral Density: The Hong Kong Osteoporosis Study. *J Clin Endocrinol Metab* **2020**, *105* (3), e619–e627. DOI: doi.org/10.1210/CLINEM/DGZ210.

91. Määttä, M.; Terho, E.; Jokinen, H.; Pulkkinen, P.; Korpelainen, J.; Heikkinen, J.; Keinänen-Kiukaanniemi, S.; Jämsä, T.; Korpelainen, R. Lifestyle Factors and Site-Specific Risk of Hip Fracture in Community Dwelling Older Women – a 13-Year Prospective Population-Based Cohort Study. *BMC Musculoskelet Disord* **2012**, *13*, 173. DOI: doi.org/10.1186/1471-2474-13-173.
